# Apatinib and fractionated stereotactic radiotherapy for the treatment of limited brain metastases from primary lung mucoepidermoid carcinoma: A case report

**DOI:** 10.1097/MD.0000000000022925

**Published:** 2020-10-23

**Authors:** Hongxia Yan, Xiaolu Li, Yi Peng, Pingping Zhang, Ning Zou, Xiyou Liu

**Affiliations:** Department of Radiation Oncology, Hubei Cancer Hospital, Tongji Medical College, Huazhong University of Science and Technology, Wuhan, P.R. China.

**Keywords:** apatinib, brain metastases, fractionated stereotactic radiotherapy, peritumoral brain edema, primary lung mucoepidermoid carcinoma

## Abstract

**Rationale:**

Apatinib is a novel anti-angiogenic agent that targets vascular endothelial growth factor receptor-2, thereby inhibiting tumor angiogenesis, and is effective in the treatment of brain metastases (BM) and peritumoral brain edema (PTBE). There are no previous reports of combination therapy with apatinib and fractionated stereotactic radiotherapy (FSRT) for BM from primary lung mucoepidermoid carcinoma (MEC).

**Patient Concerns:**

A 63-year-old man underwent left lower lobectomy and mediastinal lymph node dissection in April 2018.

**Diagnoses:**

Postoperative pathology demonstrated high-grade MEC. The patient developed 3 BM with PTBE 3 months after undergoing surgery.

**Interventions:**

The patient received a combination of FSRT and apatinib (250–500 mg/d) as maintenance therapy.

**Outcomes:**

The 3 BM showed nearly complete responses, and the PTBE areas shrank visibly. A new BM lesion occurred 7 months after the first FSRT and was treated with a second dose of FSRT. The patient developed extensive metastasis and atelectasis 9 months later. He died of pulmonary infection in December 2019. The overall survival time was 20 months.

**Lessons:**

Limited BM from primary lung MEC may be treated effectively with combination therapy with apatinib and FSRT when chemotherapy alone is not effective or tolerated. Further studies are needed to investigate the clinical outcomes and toxicities associated with the treatment.

## 1 Introduction

Mucoepidermoid carcinoma (MEC) of the lung is a type of non-small cell lung cancer (NSCLC). It is classified under the group of lung carcinoma of the salivary gland type, and it accounts for 0.1% to 0.2% of primary lung cancer. About 20% to 40% of patients with NSCLC develop brain metastases (BM).^[1]^ In the past, BM patients were typically treated using whole-brain radiation therapy (WBRT). In recent decades, stereotactic radiosurgery/fractionated stereotactic radiotherapy (SRS/FSRT) has become a widely available treatment choice. It provides for higher local tumor control while sparing more healthy brain tissue than WBRT does, decreasing the risk of detriment to the patient's neurocognitive function.^[2]^ Currently, SRS/FSRT is widely used for treating patients with 4 or more BM who have a life expectancy of more than 3 to 6 months.

Apatinib is a novel oral antiangiogenic agent that effectively inhibits tumor angiogenesis. It can induce tumor cells apoptosis in vitro and was approved as a third-line treatment for patients with gastric cancer.^[3]^ Some case reports have focused on the effects of treatment with apatinib for BM and peritumoral brain edema (PTBE) or radiation-induced brain edema.^[4,5]^ Gene mutation-targeting drugs have a good effect on BM in NSCLC with corresponding gene mutation, however, there are no particularly effective drugs for BM in primary lung MEC. In our present case, we report for the first time that FSRT and apatinib were used in combination to treat a patient with primary lung MEC who had limited BM with PTBE. The patient family gave consent for these studies and their publication, this report was approved by the Ethics Committee of Hubei Cancer Hospital, Tongji Medical College of Huazhong University of Science and Technology.

## 2 Case presentation

**Figure 1:**
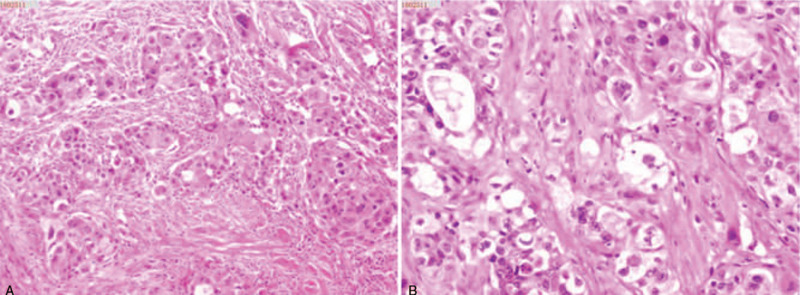
Postoperative pathology: high-grade mucoepidermoid carcinoma (A) ×200; (B) ×400.

**Figure 2:**
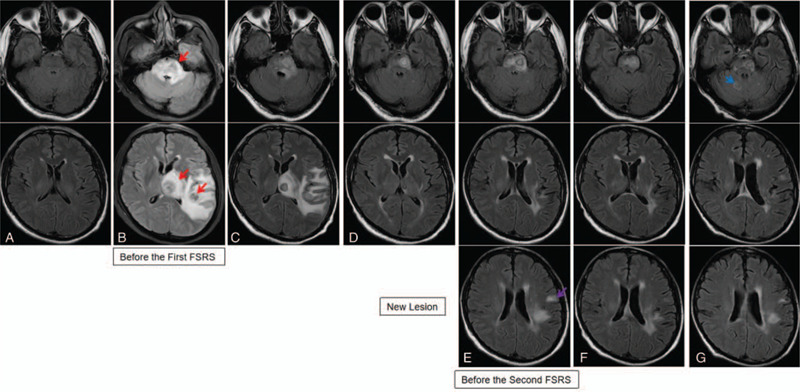
Cranial magnetic resonance imaging (MRI) scans revealed the sizes of brain metastases (BM) lesions and peritumoral brain edema (PTBE). (A) No metastatic lesion observed prior to surgery in March 2018; (B) 3 BM (red arrow indicates left temporal-parietal lobe, left thalamus lesions, and brain stem lesion), and large areas of PTBE observed in June 2018; (C) the BM lesions were reduced, and the area of the PTBE shrank progressively after radiation therapy; (D) all BM stayed reduced, and PTBE had disappeared in October 2018; (E) cranial MRI scans showed a new BM lesion in the left frontal lobe (purple arrow), and there were no obvious changes in the 3 BM in January 2019; (F) all BM lesions appeared further reduced in March 2019; (G) cranial MRI scans showed a new BM (blue arrow) in the cerebellum in October 2019.

**Figure 3:**
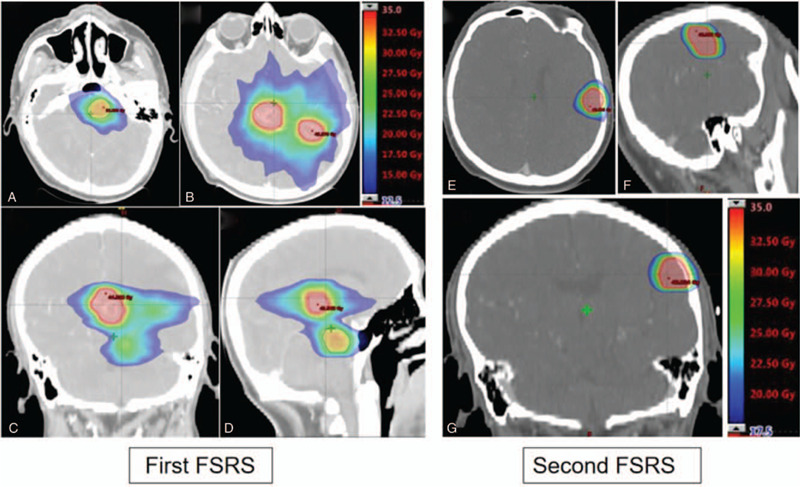
Relative radiation dose levels are indicated by the colorwash. (A-D) Relative radiation dose levels of the first fractionated stereotactic radiotherapy (dose range: 35–12.5 Gy); (E-G) relative radiation dose levels of the second fractionated stereotactic radiotherapy (dose range: 35–17.5 Gy); (A, B, and E): axial section; (C and G): coronal section; (D and F): sagittal section. Blue line indicates PTV of brain stem lesion. Red line indicates PTV of other lesions.

A 63-year-old man was admitted to our hospital because of a left lung mass and severe cough. No distant metastases were observed in the whole-body imaging examination. Cranial magnetic resonance imaging (MRI) scans showed no metastatic lesions (Fig. 2A). On April 14, 2018, the patient underwent left lower lobectomy and systematic lymph node dissection. Postoperative pathology showed high-grade MEC (Fig. 1A-B), and 2 of the left lung hilar lymph nodes were found to have metastases. Immunohistochemistry staining showed the following results: P40 (partly +), Cytokeratin (CK) 5/6 (partly +), CK7 (+), thyroid transcription factor 1 (-), NapsinA (-), CK8/18 (partly +), Calponin (partially weak +), P63 (partly +), KI-67 (40%), and Periodic Acid-Schiff (+). Genomic testing of the lung tumor revealed no common gene mutations (including epidermal growth factor receptor, anaplastic lymphoma kinase, and ROS1 [receptor tyrosine kinase encoded by the gene ROS1]). The pathological diagnosis and TNM stage were pT2N1M0 stage IIb primary lung MEC. In May 2018, the patient was treated with adjuvant chemotherapy (regimen: gemcitabine 1000 mg/m^2^, d1, d8, and cisplatin 75 mg/m^2^, d1). The patient requested that the treatment (gemcitabine at d8) be stopped because he could not tolerate the side effects. In June 2018, the patient experienced weakness, dizziness, and headache. Cranial MRI scans showed 3 BM (in the left temporal-parietal lobe, left thalamus, and brain stem) and large areas of PTBEs (Fig. 2B). He received FSRT (DT=35 Gy/5F to the left temporal-parietal lobe and left thalamus lesions, DT=25 Gy/5F to the brain stem lesion) (Fig. 3A-D). Concurrently, he received apatinib (500 mg/d). After radiotherapy, the weakness improved, and the dizziness and headache disappeared. Cranial MRI scans showed that the 3 BM had reduced, and the areas of PTBE had shrunk visibly (Fig. 2C). The response was evaluated as partial remission. In October 2018, cranial MRI scans showed that all BM remained reduced, and the PTBE had disappeared (Fig. 2D). The patient experienced oral mucositis and hypertension as side effects of apatinib treatment, and, consequently, stopped the treatment. In January 2019, cranial MRI scans showed a new BM in the left frontal lobe, and there were no obvious changes in the 3 existing BM (Fig. 2E). The new BM was treated with a second round of FSRT (DT=35 Gy/5F) (Fig. 3E-G) in January 2019. Following recovery from oral mucositis and hypertension, the patient received low-dose apatinib therapy (250 mg/d) after the second FSRT. Cranial MRI scans showed all BM appeared further reduced (Fig. 2F) in March 2019. The cough was not alleviated significantly after surgery; it became aggravated because of a severe cold, and the patient experienced hemoptysis. In July 2019, he had to stop treatment with apatinib, and received symptomatic and supportive treatment. In October 2019, cranial MRI scans showed a BM in the left cerebellum (Fig. 2G). Computed tomography imaging of the chest showed serious infection and atelectasis in the left lung. Therapy using anti-inflammatory and symptomatic treatments alone did not work. The patient and his family requested the best supportive care. On December 16, 2019, the patient died due to pulmonary infection.

## 3 Discussion and conclusion

The prognosis of low-grade MEC is excellent with 5-year survival. But for high-grade MEC prognosis is poor, and may equate that of other forms of NSCLC. In the present case, combination therapy with apatinib and FSRT had a good effect on limited BM from primary lung MEC, and had dramatic improvements in brain edema and overall survival times. Chemotherapy is a therapeutic option for BM from primary lung MEC, However, it had a poor response, and cannot be tolerated.

WBRT has historically served as the primary non-surgical therapeutic modality for BM in NSCLC.^[6]^ However, studies showed that WBRT did not enhance overall survival times in patients with a limited number of BM.^[7]^ In recent decades, SRS/FSRT has become a more popular treatment for limited BM in NSCLC than WBRT because it allows for higher local tumor control and is associated with a smaller risk of decline in neurocognitive function. However, SRS/FSRT alone as a local treatment cannot prevent distant brain recurrences.^[8]^ Combining cranial radiotherapy (SRS/FSRT) with systemic therapy using targeted agents or immunotherapy is a promising strategy to improve the outcomes of SRS/FSRT for BM in NSCLC.^[9]^

Compared to traditional chemotherapy, tyrosine kinase inhibitors (TKIs) have low molecular weights and can penetrate the blood-brain barrier. The epidermal growth factor receptor TKIs (e.g., gefitinib, erlotinib, afatinib, and particularly osimertinib) have shown promising results in the treatment of BM in patients with NSCLC who have epidermal growth factor receptor mutations by achieving response rates of 69% to 88%.^[10,11]^ Retrospective studies analyzed concurrent treatment with a target agent TKI and SRS for patients with NSCLC who have cranial and extracranial metastases. These studies have concluded that SRS can be used safely in patients receiving TKI.^[12]^ Therefore, a logical approach may be to combine target therapies with SRS, thereby overcoming the limitation of SRS in addressing micrometastatic disease in the brain and resulting in fewer distant brain failures. The postoperative pathology of this case was MEC, without driver gene mutations; there are no particularly effective drugs for BM in primary lung MEC. Consequently, we aimed to research other systematic treatments to improve patient outcomes.

BM is usually complicated with PTBE. Dehydrated drugs and steroids are effective treatments in the acute stage; however, they cannot be used for a long time. Radiotherapy may control symptoms but cannot improve edema. Studies have shown that anti-vascular drugs such as bevacizumab are effective for the treatment of brain edema. Bevacizumab, a recombinant humanized monoclonal IGG1 antibody, can reduce PTBE in glioblastoma^[13]^ and improve radiation-induced brain necrosis.^[14,15]^ Wang et al^[16]^ reported the beneficial effects bevacizumab used in combination with CyberKnife (Accuray, Sunnyvale, CA, USA) to treat patients with BM and PTBE. The patients in this study showed significant neurological improvements. However, the application of bevacizumab is limited by its high cost and adverse side effects. Apatinib is a novel tyrosine kinase inhibitor that selectively inhibits vascular endothelial growth factor receptor-2 and blocks the signal transduction of vascular endothelial growth factor binding thereby suppressing tumor angiogenesis. Some case reports have focused on the effect of apatinib in PTBE and radiation-induced brain edema. Hu et al^[4]^ reported that apatinib could shrink refractory radiation-induced brain edema in patients with NSCLC and NHL, and dramatically improve their symptoms. Song et al^[5]^ found that apatinib can shrink PTBE in a remarkably short period of time. Li et al^[17]^ reported that low-dose apatinib monotherapy showed excellent efficacy in the treatment of symptomatic BMs in 1 patient with triple-negative breast cancer. In our present case, cranial symptoms improved, and edematous areas reduced significantly 7 days after combination therapy with apatinib and radiotherapy. Thus, this treatment appeared to rapidly reduce the symptoms of brain edema. No other BM occurred during apatinib treatment; however, a new brain lesion appeared 3 months after the treatment was stopped, suggesting that apatinib therapy should be included in maintenance treatment.

Common adverse effects (AEs) of apatinib include hypertension, mucositis, proteinuria, hand-foot skin reaction, weakness, diarrhea, and rare toxicities including leukopenia, neutropenia, and thrombocytopenia. Generally, most AEs can be controlled and reversed by drug discontinuation or reduction and best supportive care.^[18]^ In the present study, hypertension and oral mucositis were observed 2 months after initiating apatinib treatment and had progressed to grade III hypertension and oral mucositis 4 months after initiating treatment. The patient stopped apatinib treatment due to grade III toxicity. One month after the withdrawal of apatinib, the AEs were alleviated. A new brain lesion was observed 3 months after stopping apatinib. Low-dose apatinib (250 mg/d) was administered to the patient after the second FSRT. The patient was administered a low dose of apatinib for 6 months and did not suffer grade III toxicity. This suggests that low-dose apatinib may be tolerated for long-term treatment. Finally, the cough persisted since the onset of surgery, and was accompanied by hemoptysis; thus, the patient was advised to stop apatinib treatment. Although this case underlined the dramatic effects of apatinib treatment of BM and PTBE, and the radiotherapy did not significantly increase the side effects of apatinib, the complications and AEs of apatinib need to be considered. This case seems to suggest that combination therapy with low-dose apatinib and RT is effective due to longer control times and tolerable AEs. Further studies are required to determine the dosage and treatment duration of apatinib in combination with SRS/FSRT for the treatment of limited BM with edema in patients with primary lung MEC.

In conclusion, we report for the first time in literature the effects of combination therapy with apatinib and FSRT in a patient with primary lung MEC who had limited BM with PTBE. While the study noted dramatic improvements in brain edema and overall survival times, there were a few AEs. This study offers a new treatment option for patients with primary lung MEC who have limited BM with PTBE; however, more clinical studies are needed to optimize the efficacy and safety of the treatment.

## Author contributions

**Conceptualization:** Hongxia Yan, Yi Peng, Ning Zou.

**Formal analysis:** Yi Peng, Pingping Zhang.

**Funding acquisition:** Ning Zou, Xiyou Liu.

**Investigation:** Hongxia Yan, Xiaolu Li.

**Methodology:** Xiaolu Li, Yi Peng.

**Project administration:** Xiyou Liu.

**Resources:** Xiaolu Li.

**Supervision:** Xiaolu Li, Xiyou Liu.

**Visualization:** Pingping Zhang.

**Writing – original draft:** Hongxia Yan.

**Writing – review & editing:** Hongxia Yan, Xiyou Liu.
